# Age-period-cohort effects in pre-existing and pregnancy-associated diseases amongst primiparous women

**DOI:** 10.1186/s13293-020-00293-9

**Published:** 2020-04-19

**Authors:** Amy Metcalfe, Sofia B. Ahmed, Kara Nerenberg

**Affiliations:** 1 Department of Obstetrics and Gynecology, Foothills Medical Centre, University of Calgary, 1403 29 St NW, Calgary, Alberta T2N 2T9 Canada; 2 Department of Community Health Sciences, University of Calgary, Calgary, Canada; 3 Department of Medicine, University of Calgary, Calgary, Canada

**Keywords:** Age-period-cohort analysis, Pregnancy, Pre-eclampsia, Gestational diabetes, Maternal age

## Abstract

**Background:**

The average age at first birth is steadily increasing in developed countries; however, demographic shifts in maternal age at childbearing have not occurred in isolation. While temporal increases in adverse pregnancy outcomes are typically attributed to increases in maternal age, little is known about how maternal health status has changed across maternal age, period of delivery, and birth cohort.

**Methods:**

Natality files were used to identify primiparous women delivering liveborn, singleton infants in the USA in 1989, 1994, 1999, 2004, 2009, and 2014 (*n* = 6,857,185). Age-period-cohort models using the intrinsic estimator adjusted for temporal trends in smoking and gestational weight gain were used to quantify temporal changes in the rates of pre-existing (chronic hypertension, pre-existing diabetes) and pregnancy-associated (pregnancy-associated hypertension, gestational diabetes, eclampsia) diseases. Log-linear models were used to model the impact of temporal changes on preterm birth, small, and large for gestational age (SGA/LGA) births.

**Results:**

Significant period effects resulted in temporal increases in the rate of chronic hypertension, pregnancy-associated hypertension, and gestational diabetes, and a significant decrease in the rate of eclampsia. These observed period effects were associated with a 10.6% increase in the rate of SGA and a 7.1% decrease in LGA. Had the rate of pre-existing and pregnancy-associated diseases remained static over this time period, the rate of preterm birth would have increased by 5.9%, but instead only increased by 4.4%.

**Conclusions:**

Independent of changes in the incidence of pre-existing and pregnancy-associated diseases as women age, the obstetric population is becoming less healthy over time. This is important, as these changes have a direct negative impact on short-term obstetric outcomes and women’s long-term health.

## Background

The increased rate of delayed childbearing in developed countries is undisputed. For example, the average age at first birth increased from 24.9 years in 2000 to 26.3 years in 2014 in the USA [1]. This rise in the average age at first birth is driven by a decrease in births to women < 20 years old (42% decrease between 2000 and 2014) and an increase in births to women ≥35 years old (23% increase between 2000 and 2014) [1]. The demographic shift towards delayed childbearing has become a significant public health issue due to the increased risks of adverse pregnancy outcomes at advanced maternal age [2]. The prevailing view is that pregnancies in women of advanced maternal age are more likely to be impacted by a pre-existing chronic disease, resulting in a reduced ability to adapt to the physiological demands of pregnancy, thus leading to an increased rate of pregnancy complications [3].

However, demographic shifts in maternal age at childbearing have not occurred in isolation. In recent decades, there have been changes in the prevalence of smoking, obesity, and other chronic diseases—all of which contribute to changes in the health status of the obstetric population and independent of the impact of changing patterns of maternal age. Age-period-cohort (APC) analysis is a classic epidemiologic approach to understand how changes in maternal age at delivery (age), population-level demographic changes (period), and early life or generational exposures (cohort) independently influence specific health outcomes [4, 5]. APC studies on obstetric outcomes have typically focused on racial differences and have not examined the impact of pre-existing or pregnancy-associated diseases on obstetric outcomes. Examining age, period, and cohort effects simultaneously is important as characteristics of older mothers have changed over time. While first births to women over 35 are now a commonplace, in previous decades, pregnancy at this age almost exclusively occurred in multiparous women having their last birth [6]. However, as the average age at first birth steadily increases, the group of primiparous women giving birth at older ages now includes more healthy women than ever before [7]. Conversely, increased use of assisted reproductive technologies now allows less healthy women, in all age groups, to conceive [8].

This study aimed to examine the age, period, and cohort effects in the prevalence of pre-existing disease and the incidence of pregnancy-associated disease in the obstetric population, and to quantify the impact that temporal changes in pre-existing and pregnancy-associated diseases over a 25-year period have had on obstetric outcomes above and beyond the impact of maternal age alone.

## Methods

Data were obtained on primiparous women delivering singleton, liveborn infants without documented congenital anomalies between 24 and 43 completed weeks of gestation according to the clinical estimate of gestational age from the United States National Center for Health Statistics (NCHS) annual natality files from 1989, 1994, 1999, 2004, 2009, to 2014 (*n* = 6,857,185). These data are obtained by the NCHS from the registration offices in all US states via the Vital Statistics Cooperative Program. Some data elements included in the birth certificate are self-reported by the mother (e.g., race, education), while others (e.g., mode of delivery, pregnancy risk factors) are directly reported by the delivery facility. Detailed instructions are provided to both mothers and health care facilities to ensure data are captured in a consistent manner. The NCHS has a robust quality assurance program in place to monitor the accuracy of data included in the birth certificates. Data were extracted on the presence of pre-existing chronic diseases (i.e., chronic hypertension, pre-existing diabetes), pregnancy-associated diseases (i.e., pregnancy-associated hypertension, gestational diabetes, and eclampsia), demographic factors (i.e., maternal age, race, educational attainment, marital status, smoking status, and gestational weight gain), and pregnancy outcomes (i.e., preterm birth, small for gestational age (SGA) at the 10th centile [9] and large for gestational age (LGA) at the 90th centile [9]). Data on pre-existing and gestational diabetes were only available following the 2003 revision of the birth certificate as prior to this point they were combined into a single variable. Only women with complete data on all variables were included in this study. As this study relied exclusively on publicly available de-identified data, it was deemed exempt from ethics review by the Conjoint Health Research Ethics Board at the University of Calgary.

### Statistical analysis

Descriptive statistics were used to characterize the population of primiparous women with pre-existing and pregnancy-associated diseases compared to their healthy peers. Chi-square tests for trend were used to examine the crude temporal trends in the prevalence of pre-existing disease and the incidence of pregnancy-associated disease over time. For the APC analysis, we first calculated the unadjusted rates of pre-existing and pregnancy-associated diseases stratified by maternal age at delivery (age), year of delivery (period), and maternal birth cohort (cohort). Due to the lack of linear independence between variables where cohort=period-age, standard regression modelling techniques are not appropriate. As such, an APC model using the intrinsic estimator (IE) was created for each outcome variable [10]. The APC_IE model uses a principal component approach to generate age, period, and cohort coefficients, standard errors, and 95% confidence intervals that represent the mean change in outcome (e.g., chronic hypertension and gestational diabetes) associated with a particular APC group relative to the mean for all APC groups combined [10]. Models were further adjusted for temporal changes in smoking and gestational weight gain. Data analysis was performed using the APC_IE procedure in Stata SE Version 14. Adjusted model coefficients were used to estimate the probability of each pre-existing and pregnancy-associated disease for each maternal age group, birth cohort, and 5-year period of delivery.

To determine the degree to which trends in the prevalence of pre-existing diseases and the incidence of pregnancy-associated diseases influenced the rate of pregnancy outcomes, we used log-linear models to examine rate ratios and rate differences for each outcome in 2004 versus 2014. This time period was chosen as information on pre-existing and gestational diabetes were only available following the 2003 revision of the US birth certificate. Crude models were originally developed including only study year; models were then adjusted for demographic variables and then further adjusted for pre-existing and pregnancy-associated diseases. The difference in the relative rates and percent change was calculated to represent the contribution of temporal changes in the prevalence and incidence of pre-existing and pregnancy-associated diseases on obstetric outcomes between 2004 and 2014 [11, 12]. For all analyses, alpha < 0.05 was considered statistically significant.

## Results

Maternal characteristics of the primiparous women are outlined in Table 1. With the exception of eclampsia, pregnancy-associated diseases (pregnancy-associated hypertension 5.4%, gestational diabetes 3.8%, eclampsia 0.5%) were more common amongst primiparous women than chronic diseases (chronic hypertension 0.9%, pre-existing diabetes 0.6%). Generally, primiparous women with a pre-existing disease were more likely to be older, married, and have graduated from high school than healthy women (Table 1). The demographic profile of women with pregnancy-associated disease was more heterogeneous. Overall, women with either a pre-existing or pregnancy-associated disease were more likely to deliver preterm or via cesarean section (Table 1).

**Table 1:** Distribution of maternal characteristics based on the presence or absence of pre-existing and gestational comorbidities

	No pre-existing or pregnancy-associated conditions (*n* = 6,322,506) % (95% CI)	Chronic hypertension (*n* = 59,730) % (95% CI)	Pre-existing diabetes^a^ (*n* = 13,400) % (95% CI)	Pregnancy-associated hypertension (*n* = 368,212) % (95% CI)	Gestational diabetes^a^ (*n* = 89,576) % (95% CI)	Eclampsia (*n* = 31,983) % (95% CI)
Maternal age						
15–19 20–24 25–29 30–34 35–39 40–44 45–49	19.3 (19.3–19.4) 30.4 (30.4–30.5) 26.2 (26.1–26.2) 17.3 (17.3–17.4) 5.7 (5.7–5.7) 1.0 (1.0–1.0) 0.1 (0.1–0.1)	9.7 (9.5–9.9) 23.4 (23.1–23.7) 28.3 (27.9–28.6) 23.1 (22.8–23.4) 12.0 (11.8–12.3) 3.3 (3.1–3.4) 0.2 (0.2–0.3)	9.7 (9.2–10.2) 25.2 (24.5–25.9) 27.7 (26.9–28.4) 22.5 (21.8–23.2) 11.7 (11.2–12.3) 3.1 (2.8–3.4) 0.2 (0.1–0.3)	21.0 (20.8–21.1) 31.1 (31.0–31.3) 26.2 (26.1–26.4) 15.1 (15.0–15.3) 5.4 (5.3–5.5) 1.1 (1.0–1.1) 0.1 (0.1–0.1)	7.8 (7.6–7.9) 22.5 (22.2–22.8) 30.2 (29.9–30.5) 25.5 (25.2–25.8) 11.2 (11.0–11.4) 2.7 (2.6–2.8) 0.2 (0.2–0.2)	27.1 (26.7–27.6) 30.9 (30.4–31.4) 22.5 (22.1–23.0) 13.0 (12.6–13.3) 5.2 (5.0–5.5) 1.1 (1.0–1.2) 0.1 (0.1–0.1)
Maternal race						
Non-Hispanic White Non-Hispanic Black Hispanic American Indian/Alaska Native Asian/Pacific Islander Other	57.4 (57.4–57.5) 12.7 (12.7–12.8) 21.0 (21.0–21.1) 0.7 (0.7–0.7) 7.0 (6.9–7.0) 1.1 (1.1–1.2)	65.2 (64.8–65.6) 21.4 (21.0–21.7) 9.0 (8.8–9.3) 0.9 (0.9–1.0) 2.8 (2.7–3.0) 0.6 (0.5–0.7)	57.6 (56.8–58.5) 17.1 (16.5–17.7) 16.8 (16.1–17.4) 1.5 (1.3–1.7) 5.7 (5.3–6.1) 1.3 (1.1–1.5)	68.3 (68.2–68.5) 15.1 (15.0–15.2) 12.5 (12.4–12.6) 1.1 (1.1–1.2) 2.4 (2.4–2.5) 0.5 (0.4–0.5)	56.6 (56.3–56.9) 10.3 (10.1–10.5) 17.5 (17.2–17.7) 0.9 (0.9–1.0) 13.5 (13.3–13.7) 1.2 (1.1–1.2)	60.1 (59.6–60.7) 20.7 (20.3–21.2) 14.5 (14.1–14.9) 1.8 (1.7–2.0) 2.4 (2.3–2.6) 0.3 (0.3–0.4)
Did not graduate high school	16.8 (16.7–16.8)	10.5 (10.2–10.7)	13.7 (13.1–14.3)	17.0 (16.9–17.1)	10.8 (10.6–11.0)	22.5 (22.1–23.0)
Not married	47.5 (47.4–47.6)	37.7 (37.3–38.1)	46.1 (45.2–46.9)	42.1 (41.9–42.2)	36.7 (36.3–37.0)	46.1 (45.6–46.7)
Current smoker	17.2 (17.1–17.2)	16.1 (15.9–16.4)	18.5 (17.8–19.1)	14.7 (14.6–14.9)	15.1 (14.9–15.3)	13.3 (12.9–13.6)
Gestational weight gain ≥40 lbs	30.0 (29.9–30.1)	28.4 (28.0–28.8)	29.2 (28.430.0)	40.0 (39.8–40.2)	22.3 (22.0–22.6)	37.4 (36.8–38.0)
Delivered via cesarean section	27.6 (27.5–27.6)	48.7 (48.3–49.1)	60.3 (59.5–61.2)	41.6 (41.4–41.7)	42.4 (42.1–42.7)	51.5 (51.0–52.1)
Small for gestational age infant (< 10th centile)	11.5 (11.4–11.5)	17.9 (17.6–18.2)	10.7 (10.2–11.2)	18.4 (18.3–18.6)	11.5 (11.3–11.7)	24.9 (24.4–25.4)
Large for gestational age infant (> 90th centile)	6.8 (6.8–6.8)	7.6 (7.3–7.8)	18.1 (17.4–18.7)	7.4 (7.3–7.5)	10.0 (9.8–10.2)	6.0 (5.7–6.2)
Preterm birth (gestational age < 37 weeks)	7.4 (7.4–7.4)	20.1 (19.7–20.4)	23.8 (23.1–24.6)	18.1 (18.0–18.3)	11.6 (11.4–11.8)	31.5 (31.0–32.0)

^a^Data on women with pre-existing and gestational diabetes is only available following the 2003 revision to the birth certificate

**Fig. 1:**
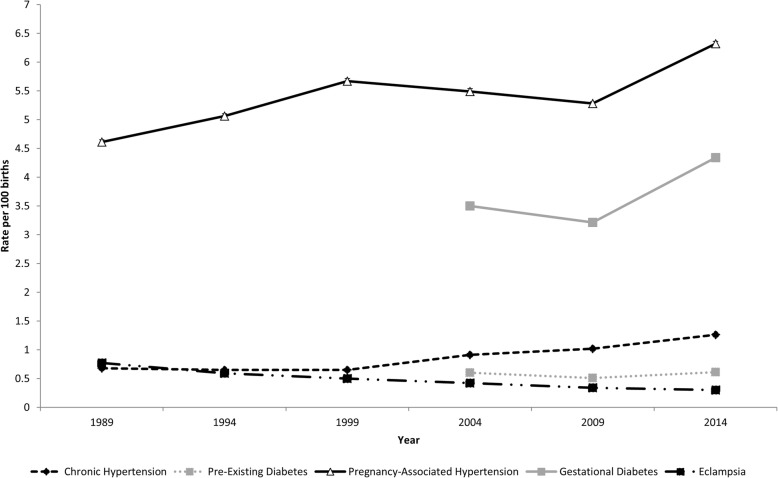
Temporal trends in the prevalence of pre-existing diseases and the incidence of pregnancy-associated diseases amongst primiparous women delivering singleton, liveborn infants in the USA

Significant temporal trends in the prevalence of pre-existing disease and the incidence of pregnancy-associated disease were observed over time (Fig. 1). With the exception of eclampsia which had a significant decrease over time (*p* < 0.001), the rate of all other conditions increased over time (*p* < 0.001). As expected, the unadjusted stratified analysis showed strong age effects for all pre-existing and pregnancy-associated diseases, with higher rates being observed in older women (Appendix 1). Of note, crude period effects were also observed. The rate of chronic hypertension, pregnancy-associated hypertension, and gestational diabetes increased over time for all age groups, while the rate of eclampsia decreased over time for all age groups (Appendix 1). For example, 30–34 years old delivering in 2014 (1.67%, 1.62–1.72) had the same prevalence of chronic hypertension that 35–39 years old had in 1999 (1.68%, 1.57–1.80). Mixed period effects were observed for pre-existing diabetes, which increased in some age groups (i.e., 15–19 years old) and decreased in others (i.e., 45–49 year olds) (Appendix 1).

**Fig. 2:**
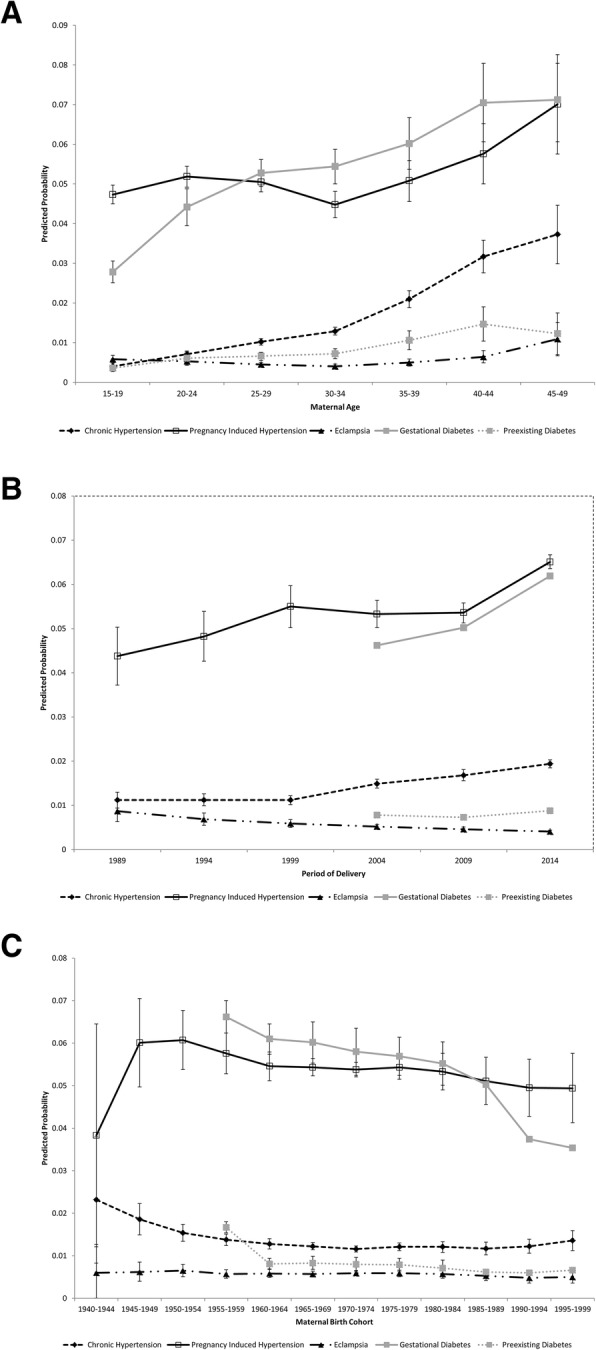
Predicted probability of pre-existing and pregnancy-associated diseases by **a** Maternal age group, **b** Period of delivery, and **c** Maternal birth cohort (based upon age-period-cohort models adjusted for temporal trends in smoking and gestational weight gain) amongst primiparous women delivering singleton, liveborn infants in the USA

Figure 2 and Appendix 2 illustrate the adjusted age, period, and cohort effects on the predicted probability of pre-existing and pregnancy-associated diseases. With regards to pre-existing diseases, significant age and period effects were observed for chronic hypertension with significantly higher predicted probabilities in older women and in more recent time periods. No significant trends were observed for pre-existing diabetes. With regards to pregnancy-associated diseases, significant age effects were observed in the youngest groups for gestational diabetes, but predicted probabilities plateaued once women reached their 30s. Significant period effects were observed for all conditions—both pregnancy-associated hypertension and gestational diabetes have significantly higher predicted probabilities in recent time periods—while the predicted probability of eclampsia has decreased significantly over time. No cohort effects were observed for any of the pregnancy-associated diseases studied.

The crude rate of SGA per 1000 births was 113.4 in 2004 and increased to 123.7 in 2014 (RR = 1.09, 95% CI 1.08–1.10) (Table 2). This increase became stronger after adjustment for demographic variables (RR = 1.13, 95% CI 1.12–1.4) and further increased after adjustment for pre-existing and pregnancy-associated diseases (RR = 1.23, 95% CI 1.22–1.25) (Table 2), indicating that the observed period effects in pre-existing and pregnancy-associated diseases are associated with a 10.6% increase in the rate of SGA. Conversely, the temporal changes in pre-existing and pregnancy-associated diseases are also associated with a 7.1% decrease in LGA (Table 2).

**Table 2:** Contribution of temporal trends in pre-existing and gestational comorbidities on perinatal outcomes

Perinatal outcome	Rate per 100 births		Relative change (2004 vs. 2014)		
	2004 (***n*** = 1,121,535)	2014 (***n*** = 1,244,642)	Rate difference (95% CI)	Rate ratio (95% CI)	Percent change
Small for gestational age					
Crude Adjusted for demographic variables^a^ Adjusted for demographic variables^a^ and comorbidities^b^ Incremental effect of comorbidities^b^	113.35 110.59 101.26	123.65 124.77 124.95	10.30 (9.48–11.13) 14.18 (13.36–15.01) 23.69 (22.88–24.50)	1.09 (1.08–1.10) 1.13 (1.12–1.14) 1.23 (1.22–1.25) 0.10 (0.10–0.11)	9.09 12.83 23.40 10.57
Large for gestational age					
Crude Adjusted for demographic variables^a^ Adjusted for demographic variables^a^ and comorbidities^b^ Incremental effect of comorbidities^b^	71.47 72.68 78.74	72.02 71.46 71.82	0.54 (− 0.12, 1.20) − 1.23 (− 1.89, − 0.57) − 6.92 (− 7.60, − 6.25)	1.01 (1.00–1.02) 0.98 (0.97–0.99) 0.91 (0.90–0.93) − 0.07 (− 0.07, − 0.06)	0.76 − 1.69 − 8.79 − 7.10
Preterm birth					
Crude Adjusted for demographic variables^a^ Adjusted for demographic variables^a^ and comorbidities^b^ Incremental effect of comorbidities^b^	86.47 83.80 84.72	89.06 88.70 88.46	2.59 (1.87–3.31) 4.90 (4.18–5.62) 3.73 (3.02–4.45)	1.01 (1.02–1.04) 1.06 (1.05–1.07) 1.04 (1.03–1.06) − 0.02 (− 0.02, − 0.01)	2.99 5.85 4.41 − 1.44

^a^Demographic variables include maternal age, maternal race, maternal education, marital status, smoking status, and gestational weight gain

^b^Comorbidities include chronic hypertension, pregnancy associated hypertension, eclampsia, pre-existing diabetes, and gestational diabetes

The crude rate of preterm birth per 1000 births was 86.5 in 2004 and increased to 89.1 in 2014 (RR = 1.01, 95% CI 1.02–1.04) (Table 2). This increase became stronger after the adjustment for demographic variables (RR = 1.06, 95% CI 1.05–1.07), but was attenuated after the adjustment for pre-existing and pregnancy-associated diseases (RR = 1.04, 95% CI 1.03–1.06). Had the rate of pre-existing and pregnancy-associated diseases remained static over this time period, the rate of preterm birth would have increased by 5.9%, but instead only increased by 4.4%, a reduction of 1.4%.

## Discussion

As anticipated, this study observed higher rates of pre-existing and pregnancy-associated diseases in older women; however, period effects were also observed in chronic hypertension, pregnancy-associated hypertension, and gestational diabetes, meaning that not only is the obstetric population getting older, but also women are less healthy over time. By controlling for temporal changes in the underlying obstetric population related to changes in maternal age, gestational weight gain, and smoking status, the temporal trends from the APC analysis more accurately estimate true changes in the incidence and prevalence of these conditions than crude temporal trends which do not reflect other changes that may be occurring in the population simultaneously (e.g., changes in clinical care practices). These matters as a recent Australian study showed that the presence of medical comorbidity is associated with more risk of morbidity and mortality than advanced maternal age itself [13], and our findings demonstrate that these temporal changes in pre-existing and pregnancy-associated diseases are impacting obstetric outcomes at the population level. This is clinically important as even the youngest age groups have an observed increase in the rate of pre-existing and pregnancy-associated diseases. While this study demonstrates the impact of these temporal changes on short-term obstetric outcomes, the increased rate of diseases in these young women may negatively influence their health outcomes as they age.

Our findings are in agreement with other studies examining age-period-cohort effects of pregnancy-associated diseases and obstetric outcomes. A study examining the APC trends in gestational diabetes in the USA from 1979 to 2010 also observed strong age and period effects in the incidence of gestational diabetes, and concluded that changing trends in the incidence of gestational diabetes were associated with temporal decreases in the incidence of LGA births [12]. Similarly, a study examining APC trends in the incidence of pre-eclampsia in the USA between 1980 and 2010 also observed both age and period effects [4].

Two studies examining APC trends in SGA in the USA between 1975 and 2000 [14] and 1989–2010 [15] both documented declines in SGA births amongst white women, and had contradictory findings amongst black women. Our study found an increasing rate of SGA amongst primiparous women that persisted after adjustment for maternal race. Our study makes a novel contribution by documenting the impact that changing rates of pre-existing and pregnancy-associated diseases has on the incidence of SGA—which may indicate that recent efforts to decrease the incidence of pre-eclampsia through the routine use of low-dose aspirin amongst high-risk women [16], may also be able to prevent the adverse neonatal sequelae associated with this diseases. As the use of aspirin for the prevention of pre-eclampsia becomes more common in routine clinical practice, it will be important to examine what impact this has on maternal and neonatal outcomes at a population level. Other studies have also reported a temporal decrease in eclampsia rates [17, 18]; a detailed review of medical records for all women with eclampsia in the Netherlands attributed this reduction to more aggressive management strategies for women with hypertensive disorders of pregnancies (i.e., increased use of anti-hypertensive medications, magnesium sulfate, and induction of labor) [17], which may also explain the findings from the current study.

This study has both strengths and limitations. The large sample size permits an investigation of rare exposures and outcomes, while the use of standard natality files with consistent reporting requirements permits an examination of temporal trends. However, this data source lacks important clinical detail on screening and treatment practices. Guidelines for prenatal screening for and treatment of pregnancy-associated diseases have changed over time meaning that our study population comprises both screened and unscreened women and potentially differences in treatments over time [12]. Changes in prenatal screening may partially explain temporal increases in pregnancy-associated diseases [12]. There is also the possibility for residual confounding by unmeasured variables such as body mass index, other health issues, and use of fertility treatments. While the US birth certificate has evolved in recent years to collect data on these important confounders, as data on these variables was not collected in older time periods, we were not able to account for their impact on temporal trends. Additionally, the US birth certificates are known to under-ascertain pre-existing and pregnancy-associated diseases [3]. As this would bias our results towards the null, the true impact of pre-existing and pregnancy-associated diseases on obstetrical outcomes may be even greater than is observed in our study. Finally, while we have attempted to mitigate the impact of changes to the birth certificate by only using variables that were consistently defined and reported on both the 1989 and 2003 versions, we cannot rule out the possibility that period effects may reflect reporting changes. However, the consistency of our findings with other studies which used different data sources supports a true period effect.

## Perspectives and significance

While the obstetric risks associated with advanced maternal age are undisputed, this study demonstrates the impact that pre-existing and pregnancy-associated diseases have on obstetric outcomes above and beyond that which can be attributed to the impact of maternal age. Importantly, the prevalence of pre-existing diseases and the incidence of pregnancy-associated diseases are increasing over time amongst primiparous women of all age groups, indicating a need to focus on preventative treatment options to optimize pregnancy outcomes and long-term women’s health.

## Data Availability

Data are available from the United States National Center for Health Statistics (https://www.cdc.gov/nchs/data_access/vitalstatsonline.htm).

## Appendix 1

Unadjusted rates of pre-existing and pregnancy-associated diseases stratified by maternal age at delivery (age), year of delivery (period), and maternal birth cohort (cohort)

Crude rate of Chronic Hypertension (%, 95% CI) by maternal age, maternal birth cohort and year of delivery

Crude rate of Pre-Existing Diabetes (%, 95% CI) by maternal age, maternal birth cohort and year of delivery

Crude rate of Pregnancy-Induced Hypertension (%, 95% CI) by maternal age, maternal birth cohort and year of delivery

Crude rate of Gestational Diabetes (%, 95% CI) by maternal age, maternal birth cohort and year of delivery

Crude rate of Eclampsia (%, 95% CI) by maternal age, maternal birth cohort and year of delivery

## Appendix 2

Estimated probabilities and 95% confidence intervals from age-period-cohort models of pre-existing and pregnancy-associated disease amongst primiparous women in the USA delivering liveborn, singleton, non-anomalies infants in 1989, 1994, 1999, 2004, 2009, and 2014 (*n* = 6,857,185)

**Table:**

	Chronic hypertension	Pre-existing diabetes	Pregnancy-associated hypertension	Gestational diabetes	Eclampsia
Maternal age					
15–19 20–24 25–29 30–34 35–39 40–44 45–49	0.0040 (0.0035–0.0044) 0.0071 (0.0063–0.0079) 0.0102 (0.0094–0.0110) 0.0129 (0.0119–0.0139) 0.0210 (0.0188–0.0231) 0.0317 (0.0276–0.0358) 0.0373 (0.0299–0.0446)	0.0036 (0.0027–0.0049) 0.0061 (0.0045–0.0077) 0.0066 (0.0055–0.0076) 0.0072 (0.0060–0.0085) 0.0106 (0.0082–0.0130) 0.0147 (0.0104–0.0190) 0.0123 (0.0070–0.0175)	0.0473 (0.0450–0.0497) 0.0519 (0.0492–0.0545) 0.0505 (0.0480–0.0531) 0.0448 (0.0415–0.0481) 0.0508 (0.0456–0.0559) 0.0576 (0.0500–0.0652) 0.0701 (0.0576–0.0826)	0.0278 (0.0251–0.0306) 0.0442 (0.0395–0.0488) 0.0528 (0.0494–0.0562) 0.0544 (0.0500–0.0587) 0.0602 (0.0537–0.0667) 0.0705 (0.0606–0.0804) 0.0712 (0.0606–0.0804)	0.0058 (0.0048–0.0068) 0.0053 (0.0043–0.0062) 0.0045 (0.0038–0.0050) 0.0040 (0.0035–0.0046) 0.0050 (0.0041–0.0059) 0.0064 (0.0049–0.0080) 0.0109 (0.0067–0.0151)
Period					
1989 1994 1999 2004 2009 2014	0.0112 (0.0094–0.0130) 0.0112 (0.0099–0.0126) 0.0112 (0.0102–0.0122) 0.0149 (0.0139–0.0159) 0.0168 (0.0156–0.0181) 0.0194 (0.0185–0.0203)	– – – 0.0078 (0.0060–0.0096) 0.0073 (0.0057–0.0089) 0.0088 (0.0078–0.0097)	0.0438 (0.0372–0.0503) 0.0482 (0.0426–0.0539) 0.0550 (0.0502–0.0597) 0.0533 (0.0502–0.0564) 0.0536 (0.0513–0.0558) 0.0651 (0.0636–0.0667)	– – – 0.0462 (0.0411–0.0514) 0.0502 (0.0454–0.0550) 0.0619 (0.0590–0.0645)	0.0087 (0.0063–0.0111) 0.0069 (0.0055–0.0083) 0.0059 (0.0050–0.0068) 0.0052 (0.0046–0.0057) 0.0046 (0.0040–0.0051) 0.0041 (0.0037–0.0044)
Cohort					
1940–1944 1945–1949 1950–1954 1955–1959 1960–1964 1965–1969 1970–1974 1975–1979 1980–1984 1985–1989 1990–1994 1995–1999	0.0232 (0.0083–0.0380) 0.0186 (0.0149–0.0223) 0.0154 (0.0134–0.0174) 0.0138 (0.0124–0.0151) 0.0128 (0.0116–0.0140) 0.0122 (0.0114–0.0131) 0.0116 (0.0109–0.0123) 0.0121 (0.0112–0.0130) 0.0121 (0.0108–0.0133) 0.0117 (0.0102–0.0132) 0.0122 (0.0106–0.0139) 0.0136 (0.0112–0.0159)	– – – 0.0167 (0.0016–0.0319) 0.0081 (0.0054–0.0109) 0.0083 (0.0066–0.0100) 0.0080 (0.0067–0.0093) 0.0079 (0.0067–0.0092) 0.0071 (0.0056–0.0087) 0.0062 (0.0046–0.0078) 0.0060 (0.0045–0.0075) 0.0066 (0.0047–0.0085)	0.0383 (0.0121–0.0645) 0.0601 (0.0497–0.0705) 0.0607 (0.0538–0.0677) 0.0576 (0.0528–0.0624) 0.0546 (0.0512–0.0579) 0.0543 (0.0523–0.0564) 0.0538 (0.0521–0.0555) 0.0543 (0.0515–0.0571) 0.0533 (0.0490–0.0576) 0.0511(0.0456–0.0567) 0.0495 (0.0428–0.0562) 0.0494 (0.0413–0.0576)	– – – 0.0662 (0.0358–0.0967) 0.0610 (0.0521–0.0700) 0.0602 (0.0548–0.0656) 0.0580 (0.0542–0.0618) 0.0569 (0.0533–0.0604) 0.0552 (0.0503–0.0600) 0.0503 (0.0447–0.0558) 0.0374 (0.0329–0.0419) 0.0354 (0.0303–0.0405)	0.0060 (0.0000–0.0127) 0.0062 (0.0040–0.0085) 0.0065 (0.0051–0.0080) 0.0057 (0.0047–0.0067) 0.0058 (0.0049–0.0068) 0.0057 (0.0050–0.0064) 0.0059 (0.0053–0.0065) 0.0059 (0.0051–0.0066) 0.0057 (0.0047–0.0067) 0.0053 (0.0042–0.0064) 0.0048 (0.0036–0.0059) 0.0050 (0.0036–0.0065)

All models are adjusted for maternal smoking and gestational weight gain.
